# Gene Therapy for Heart Failure: Impact on Mitochondrial Dysfunction

**DOI:** 10.3390/biomedicines14020344

**Published:** 2026-02-02

**Authors:** Mikhail Blagonravov, Anastasia Sklifasovskaya, Ruslan Karpov, Vera Ovechkina, Sofya Andrianova, Sergey Syatkin, Vsevolod Belousov, Andrey Mozhaev

**Affiliations:** 1 Institute of Medicine, RUDN University, 6 Miklukho-Maklaya St, 117198 Moscow, Russia; sklifasovskaya-ap@rudn.ru (A.S.); syatkin-sp@rudn.ru (S.S.); 2 Laboratory of Molecular Technologies, Shemyakin-Ovchinnikov Institute of Bioorganic Chemistry, Russian Academy of Sciences, 117997 Moscow, Russia; kruslan148@gmail.com (R.K.); vs_ovechkina@mail.ru (V.O.); skandrianova@edu.hse.ru (S.A.); belousov@fccps.ru (V.B.); a.a.mozhaev@gmail.com (A.M.); 3 Institute of Translational Medicine, Pirogov Russian National Research Medical University, 117997 Moscow, Russia; 4 Joint Department with RAS Shemyakin-Ovchinnikov Institute of Bioorganic Chemistry, Faculty of Biology and Biotechnology, National Research University Higher School of Economics, 105066 Moscow, Russia; 5 Federal Center of Brain Research and Neurotechnologies, Federal Medical Biological Agency, 117513 Moscow, Russia; 6 Life Improvement by Future Technologies (LIFT) Center, 143025 Moscow, Russia

**Keywords:** gene therapy, adeno-associated viruses (AAVs), vectors, mitochondrial biogenesis, mitochondrial dysfunction, heart failure

## Abstract

Mitochondria serve as an essential component in the maintenance of cardiac function, and targeting them may represent a promising approach to handling heart failure (HF). HF in this review refers to various etiologies, including ischemic cardiomyopathy, dilated cardiomyopathy, and hypertrophic cardiomyopathy, unless otherwise specified. Mitochondrial dysfunction, a distinctive feature of HF, leads to a progressive decrease in bioenergetic reserves due to switching of energy production from oxidation of fatty acids in mitochondria to glycolytic pathways. The main problem in developing methods to improve mitochondrial function lies in the fact that protein preparations injected through the bloodstream cannot enter cells through the plasma membrane. Modern gene therapy involving the delivery of missing genes to cells using adeno-associated virus (AAV) vectors has the potential to improve the function of cardiomyocytes (CMCs). This type of therapy aims to target proteins that have been lost, damaged, or altered due to pathological conditions in the myocardium. This review summarizes pathophysiological mechanisms associated with mitochondrial dysfunction, which is mainly caused by increased oxidative stress and impaired mitochondrial biodynamics under HF progression. It also addresses possible ways to modulate these processes using gene therapy. Special attention is paid to modern characteristics of AAVs that can be used as vectors for the efficient delivery of desired genes to CMCs.

## 1. Introduction

Targeting mitochondrial biodynamics is a promising approach for preventing and correcting heart failure (HF) progression. Currently, most medications focus on treating the symptoms of this pathological condition rather than the underlying cause of heart remodeling. Mitochondria play a crucial role in maintaining cardiac function, and their targeted intervention could be a more effective strategy for treating HF [1]. Most of the ATP used by the heart (approximately 95%) is produced through oxidative metabolism in mitochondria, utilizing free fatty acids as the primary substrate for oxidation [2]. Mitochondrial function is maintained by several closely coordinated mechanisms, which are collectively referred to as the mitochondrial quality control system. This system includes the following processes: fusion and fission, degradation, and biogenesis [3]. Mitochondrial dysfunction, a hallmark of HF, results in a progressive reduction in bioenergetic reserves due to the shift in energy production from mitochondrial fatty acid oxidation to glycolytic pathways. This process leads to a decrease in fatty acid oxidation [4]. Enhanced glucose uptake and metabolism also serve as growth signals that contribute to pathological remodeling of the heart [5]. In the later stages of HF, ketone bodies can also serve as substrates for mitochondrial oxidation [6]. As a result of changes in the oxidative substrate, oxidative stress increases, inducing cardiomyocyte (CMC) apoptosis. This, in turn, causes dysregulation of calcium homeostasis and damage to proteins and lipids. Moreover, there is a leakage of mitochondrial DNA and inflammation, which ultimately stimulates various signaling pathways leading to cardiac remodeling and HF [6]. In addition, the neurohumoral dysregulation caused by angiotensin II, endothelin-1, and sympatho-adrenergic hyperactivation that occurs in HF stimulates the hypertrophy of ventricular CMCs and exacerbates cellular damage [7].

Mitochondrial DNA (mtDNA) is a key factor in the development of HF. In a study of the left ventricle in patients with end-stage HF, it was found that the mtDNA levels decrease by more than 40%. This reduction in mtDNA leads to a significant decrease in mitochondrial replication, which in turn worsens mitochondrial biogenesis [8]. Furthermore, the depletion of mtDNA is an early indicator of left ventricular hypertrophy during the progression from hypertrophy to insufficiency in individuals with HF [9]. This loss of mtDNA is preceded by oxidative stress, which causes a decrease in the expression of genes encoded by mtDNA and complex respiratory chain enzymes [10]. It is noteworthy that this oxidative stress only affects mtDNA, but not the nuclear genome [10], which potentially arises from the lack of protective histones in mtDNA, which, as a rule, has a less pronounced epigenetic modification [11].

HF is commonly understood as a complex clinical syndrome resulting from structural or functional impairment of ventricular filling or ejection of blood [12]. The term “HF” in this review covers multiple etiologies, including ischemic cardiomyopathy, dilated cardiomyopathy (DCM), hypertrophic cardiomyopathy (HCM), and restrictive cardiomyopathy (RCM). The scope of this article primarily encompasses chronic HF with reduced ejection fraction (HFrEF)—a clinically defined HF phenotype characterized by reduced left ventricular systolic function [13,14], in which mitochondrial dysfunction is a well-established and central pathophysiological mechanism [15]. Where relevant, specific conditions are indicated in the text.

The challenge in improving the function of mitochondria within the heart lies in the fact that, when administered systemically via the bloodstream, drugs may be unable to penetrate through the plasma membrane of the cell. At the same time, intracellular protein deficiency, such as heat shock proteins, proteins and enzymes that mediate oxidative phosphorylation processes, triggers changes in CMC metabolism and the formation of “vicious pathological circles”. Modern gene therapy, based on the delivery of genes for missing molecules intracellularly through the adenovirus pathway (AAV-delivery therapy), could improve the functional state of CMCs by targeting proteins that are lost, destroyed, or change their function in response to myocardial alterations [16,17]. Repairing damaged mitochondria and removing dysfunctional ones are essential for maintaining homeostasis and preventing CMC death. Genetic forms of cardiomyopathy, such as mutations in *DES*, *LMNA*, or mitochondrial genes, can lead to mitochondrial dysfunction. These cases illustrate that mitochondrial defects are not only secondary to HF but may also result directly from pathogenic gene variants.

In this review, we will discuss the pathophysiological mechanisms associated with mitochondrial dysfunction, which mainly depend on increased oxidative stress and impaired mitochondrial biodynamics related to the progression of HF, and explore ways to modulate these processes using gene therapy.

## 2. Mitochondrial Biogenesis in HF

In HF, mitochondrial dysfunction contributes not only to energy depletion but also to disruption of ion homeostasis and an increase in ROS production. These inadequate responses exacerbate CMC stress, promote apoptosis, and accelerate pathological remodeling [4]. Oxidative stress is linked to morphological and functional abnormalities, which contribute to the development of cardiac hypertrophy and the progression of HF [18]. It should be noted that these mechanisms are involved in the pathogenesis of HF of different etiologies, including ischemic, hypertensive, and genetic cardiomyopathies and others. The general pattern of mitochondrial dysfunction appears to be consistent across all these conditions.

This decline in bioenergetic reserves occurs regardless of HF etiology, emphasizing that mitochondrial dysfunction is a convergent pathophysiological mechanism in various forms of the disease [2]. Neurohormonal abnormalities, systemic inflammation, and cellular stress contribute to mitochondrial dysfunction in HF, which is followed by an increase in the production of ROS and the release of nitric oxide synthase (NOS). Increased ROS also inhibits mitochondrial oxidative phosphorylation and can trigger mPTP opening, further reducing ATP production. These molecular processes also lead to changes in the cellular regulation of calcium exchange, contributing to cellular apoptosis, fibrosis, and hypertrophy of CMCs. These events ultimately lead to cardiac remodeling and the progression of HF [19,20].

This pathophysiological basis is shared across acquired and genetic forms of HF. In ischemic or hypertensive cardiomyopathy, neurohormonal activation and systemic inflammation suppress mitochondrial oxidative capacity. In inherited disorders, such as desminopathy (caused by *DES* mutations, OMIM #125660), Barth syndrome (BTHS, OMIM #302060) associated with a mutation in the tafazzin gene (*TAZ*, OMIM #300394), or LMNA-related cardiomyopathy (*LMNA*, OMIM #150330), structural or metabolic gene defects directly impair mitochondrial integrity, leading to mislocalization, respiratory chain deficiency, and oxidative stress [2]. It was also found that some pathogenic variants in the *DES* gene, encoding the structural intermediate filament protein desmin, were associated with mitochondrial defects in individuals suffering from familial left ventricular non-compaction cardiomyopathy (LVNC) [21]. Thus, regardless of origin, HF is mediated by mitochondrial failure as one of the common pathogenetic mechanisms.

HF is characterized by a shift in energy production from the oxidation of fatty acids within mitochondria and towards glycolytic pathways, in order to maintain adequate levels of ATP [22,23]. This metabolic shift reflects a decline in mitochondrial oxidative capacity rather than an efficient substrate adaptation. Importantly, increased reliance on glycolysis does not compensate for impaired fatty acid oxidation, as pyruvate is often inefficiently oxidized due to inhibition of pyruvate dehydrogenase and reduces Krebs cycle activity in failing CMCs. Although glycolysis produces pyruvate, which can be oxidized in mitochondria, the ATP output from glycolysis is insufficient to meet the high-energy demands of CMCs, accounting for less than 5% of total ATP, which explains why this metabolic switch does not prevent energy depletion [24]. In contrast, ketone bodies can be efficiently oxidized by mitochondria in HF, as their utilization bypasses pyruvate dehydrogenase and provides a more oxygen-efficient source of acetyl-CoA, thereby partially preserving mitochondrial ATP production under metabolic stress. Therefore, the sanogenetic mechanisms employed to restore energy balance contribute to the development of pathological pathways resulting in cardiac remodeling [25]. Increased glucose metabolism also suppresses catabolism of branched-chain amino acids (BCAAs), activating mTOR signaling and promoting hypertrophy [26]. Moreover, the inability to oxidize fatty acids can lead to the accumulation of lipotoxic metabolites [27]. In this context, various studies have demonstrated dysregulation of several molecular pathways involved in fatty acid metabolism. Data obtained from both animal and human studies suggest that the levels of peroxisome proliferator-activated receptor α (PPARα), a transcription factor that regulates the transport of fatty acids into mitochondria and peroxisomes, and the coactivator peroxisome-proliferator γ coactivator 1 α (PGC-1α), decrease with HF [28]. Additionally, elevated malonyl-CoA inhibits carnitine palmitoyltransferase 1 (CPT1), limiting fatty acid entry into mitochondria and contributing to energy deficiency [29]. These observations are consistent with findings from both animal models and human tissue studies, emphasizing the translational relevance of PPARα and PGC-1α as potential therapeutic targets. Preclinical studies demonstrate that adeno-associated virus (AAV)-mediated overexpression of *CPT1B* (OMIM, #601987) in CMCs restores the activity of fatty acid oxidation (FAO), attenuates hypertrophy, and reduces mitochondrial ROS [29,30]. Similarly, strategies to enhance sirtuin activity—either via direct AAV delivery or indirect NAD^+^ augmentation (e.g., via NMNAT or NAMPT)—could reactivate PGC-1α and improve mitochondrial quality control, though this approach remains underinvestigated in cardiac models. Notably, AAV-driven *CPT1B* expression bypasses malonyl-CoA inhibition, offering a gene-centric solution to lipotoxicity [29].

**Figure 1:**
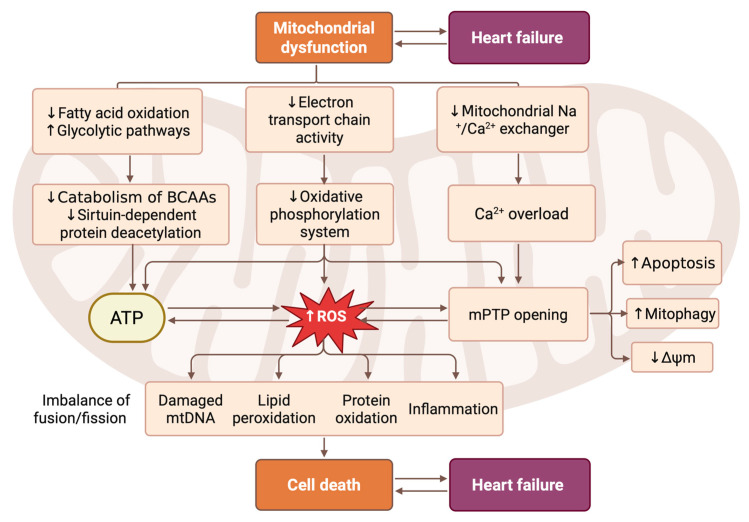
Pathophysiological cascade linking mitochondrial dysfunction to CMC death and HF progression. This scheme illustrates the central role of mitochondrial dysfunction in driving the pathogenesis of HF through interconnected metabolic, bioenergetic, and signaling pathways. Mitochondrial impairment is initiated with reduced fatty acid oxidation and increased dependence on glycolytic metabolism, accompanied by diminished electron transport chain activity and oxidative phosphorylation. This leads to decreased ATP production, elevation of reactive oxygen species (ROS) levels, and calcium overload due to impaired mitochondrial Na^+^/Ca^2+^ exchange. The resulting oxidative stress triggers lipid peroxidation, protein oxidation, mtDNA damage, and inflammation, while also promoting mitochondrial permeability transition pore (mPTP) opening. These events activate subsequent processes, including apoptosis, mitophagy, and loss of mitochondrial membrane potential (Δψm). Concurrently, imbalanced mitochondrial fusion/fission dynamics further exacerbate their dysfunction. Together, these mechanisms converge on CMC death, which directly contributes to cardiac remodeling and the clinical manifestation of HF. Created in BioRender. Abakumova, T. (2026) https://BioRender.com/l7th57b.

Increased acetylation of mitochondrial proteins involved in BCAA oxidation, pyruvate and succinate dehydrogenase activity, the malate–aspartate shuttle, the Krebs cycle, and fatty acid oxidation has been observed in HF models. This post-translational modification functionally impairs multiple oxidative pathways, thereby limiting the ability of mitochondria to oxidize both glucose- and lipid-derived substrates. This also contributes to increased susceptibility to the opening of the mitochondrial permeability transition pore (mPTP) [31,32]. Protein acetylation may be the result of an excessive concentration of short-chain acyl-CoA, which can be caused by a decrease in fatty acid oxidation or a decrease in the activity of sirtuin-dependent deacetylation of proteins [33]. Figure 1 schematically summarizes the interplay between metabolic remodeling, mitochondrial protein acetylation, redox imbalance, and mitochondrial dynamics in HF. Indeed, sirtuin activity is dependent on the level of NAD^+^, which is reduced in the presence of mitochondrial dysfunction and CMC hypertrophy. Reduced NAD+ availability not only diminishes sirtuin-dependent deacetylation but also disrupts mitochondrial redox balance, promoting oxidative stress. Under these conditions, hyperacetylation of mitochondrial proteins and increased ROS production can sensitize mitochondria to mPTP opening, despite the inhibitory effects of matrix NADH under physiological conditions [34]. Various studies have consistently demonstrated the protective effects of sirtuins on various biological processes. Specifically, sirtuins are known to activate the pAMPKa signaling pathway, increase levels of Bcl-2 protein, inhibit nuclear factor κB (NF-κB), reduce levels of phosphoprotein B kinase, regulate energy metabolism via acetylation, suppress expression of Wnt3a, and decrease levels of caspase-3 mRNA and poly(ADP-ribose) polymerase 1 (PARP-1) protein. Sirtuins protect mitochondria by deacetylating key enzymes, enhancing energy metabolism, stabilizing mPTP, and reducing oxidative stress, thus mitigating CMC dysfunction and HF progression [35].

Mitochondrial dynamics refers to the continuous process of mitochondrial fission and fusion. This process plays a crucial role in regulating mitochondrial metabolism and maintaining the intracellular environment [36,37]. Mitochondrial function is regulated by two distinct mechanisms: division and fusion. Division, facilitated by proteins such as dynamin-related protein 1 (DRP1), fission 1 (FIS1), mitochondrial fission factor (MFF), and mitochondrial division proteins 49 and 51 (MiD49/51), leads to mitochondrial fragmentation, which is essential for cell division and the removal of damaged mitochondria. Fusion, organized by mitofusins 1 and 2 (MFN1/2) and optic atrophy protein 1 (OPA1), results in the formation of interconnected networks that facilitate the maintenance of mitochondrial DNA integrity through complementation and selective removal of damaged mitochondrial DNA and energy distribution. While cleavage proteins primarily contribute to morphological changes, fusion proteins play various roles in mitophagy, apoptosis, and energy metabolism [38] (Figure 1).

Recent studies suggest that modulating MFN2/OPA1 balance could restore mitochondrial connectivity and improve ATP supply in HF. This provides a basis for gene therapy interventions targeting these fusion/fission proteins. In particular, these pathological processes disrupt intracellular energy metabolism through changes in substrate utilization, leading to substrate hypoxia and further compromising mitochondrial function. This hypoxia is exacerbated by the accumulation of ROS, which primarily arises as a consequence of impaired electron transport and inefficient oxidative phosphorylation, further leading to energy depletion and inhibition of mitochondrial ATP synthesis. The accumulation of ROS also triggers processes of mitophagy, which can lead to an imbalance in mitochondrial fusion and fission. This imbalance can activate programmed cell death processes and further exacerbate the severity of HF.

Remarkably, AAV-based interventions can reconfigure this system. In a murine aortic stenosis model, AAV9-mediated overexpression of *ADRB3* (OMIM #109691) restored fission/fusion equilibrium, reduced oxidative stress, and halted HF progression—without directly targeting mitochondrial machinery, but via upstream signaling modulation [39].

Altered substrate utilization in HF leads to substrate hypoxia, further impairing mitochondrial function. Accumulated ROS exacerbates energy depletion, triggers mitophagy, and may disrupt the balance between fission and fusion, promoting cell death and aggravating HF.

Mitochondria in adult CMCs can be classified into three main categories based on their location and function. The majority of mitochondria, known as interfibrillar mitochondria (IFM), are situated adjacent to myofibrils and play a crucial role in calcium signaling and providing energy for contraction. A smaller subset, sarcoplasmic submembrane mitochondria (SSM), is positioned near the membrane of subcoronary arteries and serves to supply energy for ion channel activity and signal transmission within cells. Finally, perinuclear mitochondria (PNM) are located close to the nucleus and contribute to energy requirements for transcriptional processes [40,41,42]. IFM supplies energy for contraction and calcium signaling, SSM supports ion channels and signal transduction, and PNM supports transcriptional processes. Recent research has demonstrated that, despite their distinctive structure, mitochondria are able to alter their shape in adult CMCs [43,44,45,46]. Although various observation techniques allow us to determine the dynamics of mitochondria within CMCs, the findings obtained differ from the fundamental dynamics of these organelles derived from cultured cells.

Additional research on heart tissue is therefore required in order to investigate the dynamics of mitochondrial activity in cardiac and mature CMCs. This will involve assessing the morphological condition of the myocardium, considering the characteristics of the tissue rather than individual CMCs.

Mitochondrial health in cardiac muscle cells is maintained not only through mitochondrial dynamics, but also through mitophagy (the selective isolation of damaged mitochondria through autophagy). Drp1 (dynamin-related protein 1) plays a crucial role in inducing mitophagy. Mitochondrial division mediated by Drp1 is essential for the initiation of mitophagy within the heart [47,48]. The *Drp1* C452F mutation in mice results in increased activity of the Drp1 GTPase, as well as resistance to oligomer degradation. This, in turn, leads to impaired mitophagy, mitochondrial depolarization, abnormal calcium handling, reduced ATP production, and activation of a sterile myocardial inflammatory response [49]. In mice with HF caused by transverse aortic constriction (TAC), mitophagy associated with Drp1 is initially activated and then suppressed in the heart in response to pressure overload [50]. In a rat model of HF, CMCs demonstrate increased production of ROS from the mitochondria. It is worth noting that the levels of expression of Mfn2 and Drp1 have been reduced by approximately 50%. This reduction in regulation leads to an accumulation of Parkin in the mitochondria and the subsequent induction of mitophagy. The observed regulation of mitochondrial and mitophagy dynamics mediated by Mfn2 and Drp1 appears to play a crucial role in cardiac protection, possibly through the modulation of ketone body metabolism [51]. A patient with HF with reduced ejection fraction (HFrEF) has elevated levels of Drp1. In terms of clinical relevance, Mfn2 levels in samples from patients with HFrEF remain stable [52]. Therefore, therapeutic strategies aimed at restoring mitochondrial metabolic flexibility, redox balance, and quality control may represent a rational approach to improving cardiac energetics and attenuating the progression of HF [53].

## 3. Mitochondrial Targets for Viral and Nonviral Vectors in CMCs to Reduce HF

Currently, methods of gene therapy for cardiovascular diseases, including HF, which focus on regulating the expression of the relevant genes, are being actively developed. In particular, it is possible to use the delivery of exogenous genes in order to increase the production of the corresponding proteins in case of their deficiency, as well as repairing mutant sequences to correct defective proteins [54]. These problems can be addressed through two strategies: the development of efficient methods to deliver genetically encoded tools to target cells and the search for specific molecular genetic targets that represent the key mechanisms of pathogenesis.

It is important to emphasize that the choice of both gene targets and delivery strategies should correspond to the stage of HF and the type of mitochondrial dysfunction. Recent studies show that early intervention targeting mitochondrial biogenesis or fusion/fission proteins can provide better functional results than late-stage interventions. A number of pieces of evidence indicate that mitochondrial abnormalities in HF develop depending on the stage, which, to a certain extent, determines the effectiveness of targeted gene therapy. In the early and compensated stages of HF, mitochondrial dysfunction is characterized primarily by a disorder of mitochondrial biogenesis; a change in substrate utilization: switching metabolism from fatty acid oxidation to glycolysis; and early disturbances in mitochondrial dynamics [4,55]. At this stage, the delivery of genes aimed at restoring mitochondrial biogenesis and oxidative metabolism, such as activation of the PGC-1a–TFAM axis, increased NADH/sirtuin signaling (SIRT1/SIRT3) or modulation of fusion–division balance (OPA1, MFN2), may have significant potential to preserve contractile function and to delay pathological remodeling [56,57,58,59,60]. In contrast, advanced HF with reduced ejection fraction is associated with irreversible structural remodeling, accumulation of dysfunctional mitochondria, disrupted mitochondrial quality control, and increased oxidative stress, contributing to energetic failure and cardiomyocyte loss [55]. At this stage, metabolic reprogramming strategies alone are often insufficient. However, interventions aimed at calcium handling (e.g., SERCA2a, S100A1), mitochondrial quality control, and redox balance can provide partial functional improvement, but rarely reverse disease progression [4,51]. These limitations may explain why some promising mitochondrial or metabolism-related targets have shown significant efficiency in preclinical models but have not reached the late stage of clinical trials. In the end-stage HF, mitochondrial dysfunction is characterized by profound bioenergetic failure, extensive cardiomyocyte loss, and replacement fibrosis [55]. Gene therapy approaches aimed at restoring mitochondria are unlikely to improve myocardial function if they are not combined with antifibrotic, regenerative and cellular methods [61,62]. These observations suggest that successful clinical translation of mitochondrial gene therapy requires not only target specificity, but also an accurate temporal correspondence between the selected molecular pathway and the stage of HF progression.

In addition to stage-dependent effects mentioned above, recent data indicate that sex differences in mitochondrial function can affect both the natural course of HF and the effectiveness of targeted mitochondrial interventions. Preclinical studies demonstrate that basal mitochondrial gene expression and mitochondrial DNA content differ in male and female hearts, potentially contributing to differences in diastolic function and predisposition to HF phenotypes such as HF with preserved ejection fraction (HFpEF) [63]. It was also shown that experimental mouse models of PGC-1α deficiency exhibit dilated HF accompanied by disorders of aerobic and anaerobic metabolism, calcium handling, cell structure, electrophysiology and gene expression, but structural and functional changes in female mice develop earlier and are more profound. These findings emphasize the role of key pathways of mitochondrial biogenesis in the progression of HF with PGC-1α as an essential factor of sex-specific differences in cardiac function [64]. Mitochondrial pathways and NADH/sirtuin-dependent signaling are regulated by sex hormones, as evidenced by different expression of Sirt1 and Sirt3 in aging female (versus male) hearts, which may alter oxidative protection and inflammatory reactions related to the pathogenesis of HF [30]. These gender-dependent variations suggest that optimal gene therapy strategies, including the selection of targets such as biogenesis regulators, redox mediators, and calcium processing modulators, may require sex-specific optimization and stratification in preclinical studies and further clinical translation.

AAV, which is characterized by a diverse range of serotypes exhibiting tissue-specific tropism, can be employed as a vehicle for delivering the required sequences [65]. With regard to CMCs, the most suitable viral vectors are AAV serotypes 1 [66] and 9 [67]. However, the efficiency of transduction largely depends on the method of administration. In particular, when delivered intravenously, a comparison of different serotypes of viral vectors, including AAV1, 2, 6, and 8, showed that AAV9 and AAV8 are the most efficient [68]. In case of direct intracardiac administration, AAV6 shows maximum efficacy, significantly higher than AAV9 [69]. But the intravenous method of administration is accompanied by off-target effects. It is also possible to use non-viral vectors, in particular, lipid and modified nanoparticles [54]; however, the transduction of CMCs with AAVs is significantly higher [70].

Now let us turn to the potential molecular targets for HF gene therapy. By the present time, the possibility of introducing exogenous genes into CMCs to improve the LV contractile function in HF has been investigated [71]. In particular, activation of Ca^2+^-ATPase *ATP2A2* (OMIM #108740), which encodes the cardiac isoform SERCA2a, is a promising approach to restore calcium homeostasis and reduce ventricular arrhythmias [72,73].

Although *ATP2A2*, *S100A1* (OMIM #176940), *ADRB3* (OMIM #109691), and *CAV3* (OMIM #601253) are not strictly mitochondrial proteins, they are considered “mitochondrial targets” in a functional sense because their modulation directly or indirectly affects mitochondrial bioenergetics, calcium handling, and redox homeostasis in CMCs.

Being associated with the mitochondrial dysfunction described above, these alterations result in dysregulation of calcium homeostasis, oxidative stress, protein toxicity, and ultimately CMC death [2,8].

Recent advances in AAV-mediated gene therapy have enabled precise targeting of mitochondrial dysfunction in HF. Current research is primarily focused on addressing specific disorders, such as impaired fatty acid oxidation, fragmented mitochondrial networks, or abnormal calcium regulation, using the method of cardiac-directed gene delivery. The following examples highlight possible “points of application” for AAV-mediated gene delivery to restore mitochondrial properties by modulating critical mechanisms of energy production, mitochondrial dynamics and quality control, thereby halting or reversing HF progression in experimental animal models (Table 1).

On a mouse model of HF provoked by aortic stenosis, it was shown that selective overexpression of *ADRB3* through AAV9-NDUFS6 injection led to a restoration of the mitochondrial dynamics, prevented fragmentation of mitochondria and hindered the progression of HF [39]. Using the same model of HF, the possibility of *CPT1B* gene transfer with AAV was studied. *CPT1B* is a gene encoding carnitine palmitoyltransferase 1B, which catalyzes the rate–limiting step of the carnitine shuttle necessary for the oxidation of fatty acids. It was found that overexpression of *CPT1B* in neonatal rat CMCs prevented phenylephrine-induced hypertrophy and led to a decrease in the production of reactive oxygen species in mitochondria [74].

Interestingly, the timing of gene delivery appears critical, as neonatal interventions in neonatal animals showed higher efficacy than in adult ones. Administration of AAV9-NDUFS6 to newborn mice with mitochondrial cardiomyopathy caused by a deficiency of the *Ndufs6* gene, encoding mitochondrial complex I, also hindered the development of the contractile dysfunction of the heart: the activity of the mitochondrial complex I and the structure of the cristae were sustained, and the severity of fibrosis decreased. However, such an effect was not observed in adult individuals with fully developed HF. On the contrary, mitochondrial abnormalities and increased CMC apoptosis were observed [75]. This highlights the importance of early intervention in mitochondrial gene therapy. Adult hearts with established HF may require combined strategies targeting both mitochondrial repair and mitigation of fibrosis or hypertrophy.

In some earlier studies, on a pig model of HF caused by focal ischemia of the LV, it was found that the delivery of AAV9-S100A1 (Ca^2+^-sensor protein) through the coronary veins resulted in the restoration of normal S100A1 expression and was accompanied by the normalization of calcium metabolism in CMCs, improvement of the endoplasmic reticulum state and energy metabolism [76]. It was also shown in cultures of CMCs isolated from the myocardium of patients suffering from severe congestive HF that genetically targeted therapy employing the human *S100A1* cDNA resulted in normalization of S100A1 levels. This led to an improvement in compromised mitochondrial function and the restoration of the phosphocreatine/adenosine triphosphate ratio [77].

Mitochondrial dysfunction is of particular importance in the pathogenesis of diabetic cardiomyopathy. In particular, a defect in the *NDUFA10* (subunit of mitochondrial complex I, OMIM #603835) plays a key role in the development of this pathology. A potential role of caveolin 3 (CAV3) was investigated on a mouse model of diabetic myocardial injury. The authors performed an experiment in which animals were injected intravenously with an AAV9 vector expressing *CAV3* (AAV9-CAV3) under the control of the cTnT promoter or control virus. The resulting overexpression of CAV3 attenuated mitochondrial dysfunction caused by the *Ndufa10* defect. Particularly, CAV3 interacted with NDUFA10, reducing the degradation of the lysosomal pathway in NDUFA10, restoring the activity of mitochondrial complex I, and improving mitochondrial function [78].

Taken together, these studies suggest that mitochondrial dysfunction is not only a mechanism of HF but could also be considered as a “pathogenetic target” for gene therapy. Although *CPT1B*, *ADRB3*, *S100A1*, and *NDUFS6* have shown high efficacy under certain conditions, the final outcome largely depends on the choice of vector, delivery route, disease stage, and underlying etiology (e.g., pressure overload, ischemia, or metabolic cardiomyopathy). Notably, interventions effective in neonatal or early-stage HF (e.g., AAV9-NDUFS6) may fail in advanced disease, underscoring the need for accurate timing and stratification of patients. Future efforts should prioritize the identification of novel mitochondrial targets—particularly those that return CMC metabolism to fatty acid oxidation—and integrate them into next-generation AAV platforms with enhanced cardiac specificity and immune protection.

## 4. Modern Characteristics of AAV Vectors for Cardiomyocytes

Adeno-associated viruses (AAVs) represent an effective delivery tool in clinical gene therapy. AAV is a non-enveloped, single-stranded DNA virus belonging to the *Dependovirus* genus of the *Parvoviridae* family [79]. The wild-type AAV serotype genome was first successfully cloned in the 1980s, establishing the foundational template for subsequent recombinant AAV (rAAV) vectors [80,81]. The widespread use of rAAVs is attributed to a number of factors. Firstly, the ability of AAVs to infect both dividing and non-dividing cells makes them particularly suitable for targeting post-mitotic cells such as neurons and CMCs [79]. Secondly, AAVs exhibit favorable safety characteristics, including non-pathogenicity, a low frequency of host genome integration, and the capacity for long-term episomal transgene persistence in non-dividing cells. These characteristics are coupled with the inherently high transduction efficiency of certain serotypes [82]. Furthermore, different AAV serotypes exhibit varied cellular and tissue tropism, a trait that is predominantly determined by genomic variations located within the variable regions of the viral capsid, particularly the VP3 domain [83]. For cardiac gene transfer, AAV1, AAV6, and AAV9 have emerged as the most efficacious serotypes, demonstrating successful therapeutic gene delivery to the myocardium in both rodent and large animal models.

It is important to note that while experimental studies on animal models demonstrate efficient myocardial transduction, the results can still vary depending on the species, age, and stage of HF. Careful consideration of these factors is necessary when translating these findings to clinical settings.

AAV1 is one of the most studied and the first viral vector that was approved for the use in gene therapy [84]. This serotype demonstrates one of the broadest tropism profiles among all AAV serotypes, which has led to its widespread use in preclinical and clinical research. AAV1 has been identified as the most efficient serotype for gene delivery to skeletal muscle cells in various animal models [85,86,87], yet it has also demonstrated efficient cardiac transduction and outperforms AAV2 in large-animal myocardium, particularly in case of intramyocardial delivery [88], endothelial and vascular smooth muscle cells [89], and neurons [90].

Another popular serotype for gene delivery is AAV6. This serotype is a natural hybrid resulting from recombination between AAV1 and AAV2 [91], which possesses broadened tropism and enhanced efficiency compared to its parent strains due to its hybrid receptor profile [92]. Owing to the characteristics, AAV6 demonstrates exceptional efficacy in transducing a wide range of tissues including CMCs in various animal models—from mice to sheep [93,94,95]. Moreover, in mice AAV6 demonstrated significant heart expression reaching the peak level with luciferase enzyme activity (7.9 × 10^5^ ± 3.5 × 10^5^ TF (total flux (photons/sec/cm^2^/sr)) on day 21 after indirect intracoronary injection which was the highest value among AAV1-9 serotypes [93]. Using immunohistochemical assay, it was also shown that up to 100% of CMCs in the LV anterior wall were transduced after recirculating delivery of scAAV6 [95].

Discovered in 2004, the AAV9 serotype has emerged as a leading vector for systemic gene delivery, distinguished primarily by its exceptional tissue tropism [91,96,97]. It demonstrates superior transduction efficiency in critical tissues such as cardiac and skeletal muscle, liver, and pancreas [87]. In mice, systemic delivery of rAAV9 achieves transduction of over 80% of CMCs at a dose of 1.0 × 10^11^ vg/mouse [98]. Broad targeting capability of this serotype is mediated by its unique receptor profile: it utilizes terminal N-linked galactose as a primary receptor and is hypothesized to interact with a putative integrin, employing the laminin receptor (LR) as a coreceptor. A groundbreaking characteristic of AAV9 is its ability to cross the blood–brain barrier, enabling efficient gene delivery to central nervous system cells, including neurons and astrocytes [99,100]. This capability has established AAV9 as a prime candidate for treating neurodegenerative disorders including spinal muscular atrophy and Parkinson’s disease. Another capacity of AAV9 to cross the vascular endothelium is a key factor in its enhanced cardiac targeting [101]. An additional significant advantage of AAV9 is its low immunogenicity. The prevalence of pre-existing antibodies against AAV9 in the human population is considerably lower than for other serotypes, thereby expanding the potential patient population eligible for gene therapy [102].

While the low immunogenicity of AAV9 is an advantage of this serotype, pre-existing neutralizing antibodies (nAbs) can still limit efficacy. The presentation of AAV antigens by antigen-presenting cells (APCs), primarily dendritic cells, to CD4+ T-helper cells serves as a key signal for initiating the B-cell response, which includes proliferation, affinity maturation, isotype switching, and ultimately, the production of antibodies against the viral capsid [103]. The prevalence of pre-existing anti-AAV nAbs in the human population varies from 2% to 60% depending on the serotype, with antibodies against AAV1 and AAV2 being the most common [104]. The recognition of the AAV capsid or the introduced transgene by the innate immune system activates signaling pathways, such as NF-ĸB, leading to the upregulation of major histocompatibility complex (MHC) genes and the secretion of pro-inflammatory cytokines or type I and III interferons [105]. The released mediators consequently activate genes that suppress viral replication and stimulate the development of the adaptive immune response. Another crucial mechanism is TLR-mediated signaling, primarily via TLR9, which contributes to immune responses against both the transgene and the capsid [106]. TLR9 signaling in APCs leads to antigen presentation on MHC class I and the activation of an AAV-specific cytotoxic CD8+ T-cell response [107,108]. Effector capsid-specific CD8+ T cells migrate to target tissues, where they can induce cell death through the secretion of perforin and granzymes, leading to the loss of the therapeutic transgene and inflammatory tissue damage [108,109]. However, a significant cytotoxic CD8+ T-cell response to the AAV capsid is predominantly observed in the liver [110], in contrast, muscle tissue demonstrates a notable resistance to the elimination of transgene-expressing cells [98,111]. Furthermore, AAV capsids can directly activate the alternative complement pathway, leading to the generation of anaphylatoxins C3a and C5a, opsonization of particles by C3b, and potentially, the formation of the membrane attack complex [112]. Several strategies to mitigate immune responses to AAV vectors, including methods to induce immune tolerance, are currently under investigation. One approach involves transient immunosuppression, which has been explored in multiple studies [113,114]. Another strategy focuses on the use of engineered capsids or capsids with low seroprevalence, aiming to reduce pre-existing immunity [115,116]. Additionally, optimizing the vector dose has been proposed as a way to minimize immune activation while maintaining therapeutic efficacy [117]. Finally, several approaches aim to induce immune tolerance to either the transgene or the capsid itself. They include mechanisms mediated by regulatory T cells, which have shown promising results in a number of studies [118,119].

**Figure 2:**
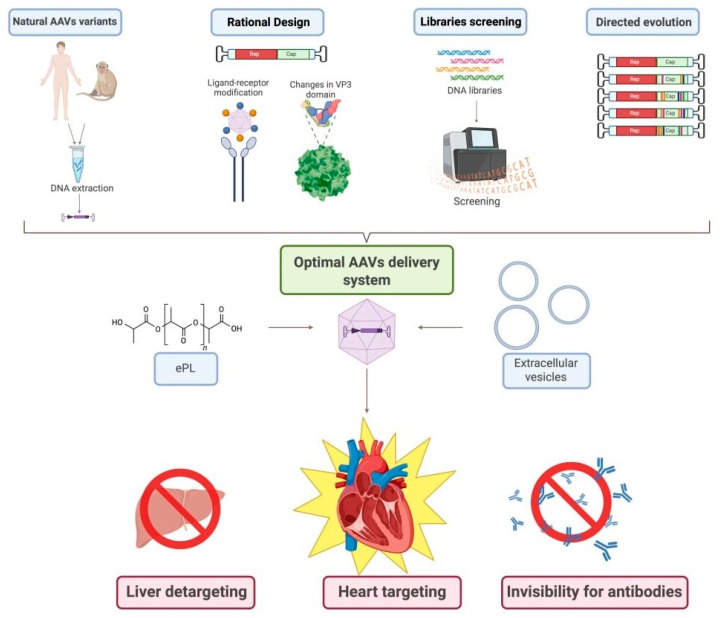
Algorithm for developing the most effective pathway for CMC transduction with AAVs. This scheme illustrates the multi-faceted approach to engineering next-generation AAV vectors with enhanced CMC specificity and reduced off-target effects, outlining four primary strategies: (1) isolation and use of natural AAV variants from human or non-human primate tissues followed by DNA extraction and cloning; (2) rational design involving structure-guided capsid modification, including ligand-receptor engineering and targeted mutagenesis of the VP3 domain; (3) high-throughput screening of diverse capsid DNA libraries using platforms such as flow cytometry or next-generation sequencing to identify clones with desired tropism and transduction profiles; and (4) directed evolution through iterative rounds of mutagenesis and selection to generate novel capsids with improved characteristics. The resulting optimized AAVs can be further refined into an advanced delivery system via encapsulation in extracellular vesicles (EVs) or complexation with polymers like ethylenediamine-poly-L-lysine (ePL) to enhance stability, cellular uptake, and immune evasion. The ultimate goal of this integrated pipeline is to achieve simultaneous liver detargeting (minimizing hepatic transduction and associated toxicity), heart targeting (maximizing transduction efficiency in CMCs), and invisibility to pre-existing neutralizing antibodies—thereby improving safety, efficacy, and clinical translatability of mitochondrial-targeted gene therapy for HF. Created in BioRender. Abakumova, T. (2026) https://BioRender.com/yogxpfu.

To improve the efficiency of CMC transduction and advance the clinical application of viral therapies, ongoing research is focused on identifying and developing novel serotypes. These strategies involve identifying new vectors or altering the capsid proteins of existing AAVs to achieve a combination of cardiospecificity and cardiotropism. An example of viral engineering is the development of the chimeric vector AAV2i8, achieved by substituting a hexapeptide in the receptor domain of AAV2 with a sequence derived from AAV8 [120]. The resulting chimeric vector inherited ability of AAV8 for efficient transduction of muscle tissue, including cardiac tissue, while demonstrating significantly reduced accumulation in the liver after systemic administration. Libraries screening of capsids and subsequent directed evolution also is frequently employed to select variants that transduce CMCs most effectively. For example, MyoAAV 1A/2A has been identified as demonstrating record-breaking efficiency in delivering genes to muscles [121]. Furthermore, the process of random mutagenesis of surface-exposed regions of the AAV9 capsid protein not only enabled the creation of new variants of AAV9.45 and AAV9.61 with selectively reduced tropism for the liver, but also identified key sites responsible for liver tropism [122] (Figure 2).

These engineering approaches directly address concerns regarding off-target transduction and vector loss in peripheral tissues. Optimizing capsid design is critical for maximizing myocardial specificity while minimizing systemic exposure.

Efficacy of myocardial transduction by various AAV serotypes demonstrates significant dependence on delivery methods and dosage. In general, AAVs delivery during the gene therapy can be performed via two ways: surgical and percutaneous techniques [82]. The main surgical technique involves the injection of medication into the heart muscle using thin needles [123] achieving local transduction rates up to 60% of CMCs in dog models after scAAV6 percutaneous transendocardial delivery [124]. This technique offers distinct advantages, including high local vector concentration, circumvention of the endothelial barrier, and minimized off-target organ exposure. However, its applicability is limited by the restricted diffusion radius from the injection site, making it unsuitable for treating diffuse cardiac pathologies, for example, HF [124].

To increase efficiency of transduction, a “gene painting” technique was developed. The method employs direct epicardial application of AAV vectors combined with poloxamer gel to prolong viral contact time and mild trypsinization to enhance viral penetration [125]. This methodology enables targeted gene delivery to atrial tissue with high efficiency [126]. However, both this approach and intramyocardial injection constitute invasive procedures that may precipitate various associated adverse effects, including inflammatory responses and procedural complications.

In addition, researchers are using molecular cardiac surgery with recirculating delivery (MCARD) [127]. This approach is an invasive but extremely effective way of delivering genetic constructs, where the animal is connected to an artificial circulatory system [128]. It enables the cardiac circulation to be completely isolated from the systemic circulation, ensuring that blood containing the vector recirculates exclusively through the coronary system. The primary benefits of MCARD include enhanced myocardial contact time through prolonged intracoronary vector circulation, protection from pre-existing antibody neutralization via the closed-circuit system, and minimized risk of off-target transduction. These findings support the concept that delivery method critically influences therapeutic outcomes. Less invasive but equally efficient delivery techniques remain a major area of active research.

An alternative strategy is transvascular delivery, which involves vector administration into the vascular compartment, and subsequent migration to CMCs. Systemic intravenous administration is the simplest method but the least effective, as it is characterized by massive vector loss in peripheral tissues [129]. Rodents can be effectively infected with AAVs through intravenous administration, but this is much more difficult to achieve in larger animals, and in humans [130].

Antegrade intracoronary injection is the least invasive method of delivering genetic constructs to the myocardium and is widely used in preclinical and clinical studies of HF [131,132,133]. This strategy facilitates the homogeneous distribution of the virus throughout the myocardial tissue; nevertheless, its CMC transduction efficiency remains significantly restricted [130,134]. An alternative strategy to enhance transduction efficiency is retrograde infusion, which involves injection of the viral vector directly into the coronary vein [135]. The contact of the vector with the coronary endothelium is prolonged, which enhances its passage into the tissue, and the increased intravascular pressure increases the proportion of transduced CMCs [136].

For comparison, in rodent models, AAV1 and AAV6 demonstrate optimal efficiency with intramyocardial or intracoronary injection, whereas AAV9 is most suitable for transvascular delivery. The proportion of transduced CMCs is also dose-dependent. At high viral loads, all cardiotropic serotypes exhibit comparable transduction efficacy; however, at medium doses, only AAV9 maintains high myocardial transduction activity [137]. A different trend can be seen in large animal models. Intracoronary administration demonstrates superior efficacy for AAV6 [138]. Systemic intravenous delivery is more complex: while AAV9 achieves substantial skeletal muscle transduction but limited myocardial expression, AAV8 shows robust cardiac transduction [139]. Notably, AAV9 demonstrates superior delivery efficiency in sheep models using MCARD [140]. Therefore, optimal serotype choice in large animals is context-dependent, dictated by the delivery method, dosage, and the specific animal model. To summarize available data, Table 2 provides an overview of cardiac transduction efficiency of AAV serotypes depending on the animal model, administration method, and vector dose.

Overall, these data emphasize that both vector selection and delivery route are critical determinants of therapeutic success. Interspecific variability highlights the need for thorough preclinical testing before switching to HF therapy in humans.

## 5. Conclusions and Perspectives

Despite significant progress in the development of gene therapy approaches for the treatment of HF, which are aimed at correcting mitochondrial dysfunction, there are still a number of limitations and challenges that require attention in future research.

Firstly, there are some limitations on the tropism and efficiency of delivery of AAV particles to target cells and tissues in the body. For example, serotypes AAV6 and AAV9 with a high degree of tropism for CMCs have varying degrees of transduction efficiency depending on the method of delivery, the stage of HF development, concomitant pathologies, the type of experimental animal, or individual characteristics of the organism. From a clinical point of view, intravenous administration of the vector is preferable due to the reduced risk of adverse effects, but there is a significant loss of the vector in peripheral tissues, such as the liver, which leads to a decrease in AAV delivery to the myocardium. At the same time, more effective invasive methods of delivering viral vectors to the myocardium, such as MCARD, are not suitable for widespread use in clinical practice. Secondly, even when using AAV9 with low immunogenicity, the injected viral particles may be neutralized by circulating pre-existing antibodies to AAV capsids in the majority of the human population. In addition, successful transduction of CMCs may lead to the development of cellular immunity directed against transgenic CMCs. Thirdly, the effectiveness of transduction may decrease depending on the severity of HF in patients. For example, in conditions of pre-existing extensive cardiosclerosis, severe ischemic cardiomyopathy, hypertrophy or myocardial remodeling, which alter coronary blood flow and tissue structure, contributing to a reduction in successfully transduced functional CMCs. As demonstrated in the treatment of *Ndufs6* deficiency, therapeutic intervention was effective only in neonatal HF but had no effect on HF in adult subjects.

Despite the convincing effectiveness of preclinical models, the clinical translation of cardiac gene therapy has faced significant difficulties, especially in the CUPID 2 trial of AAV1-mediated *ATP2A2* (*SERCA2a*, OMIM #108740) delivery for advanced HF. While the earlier CUPID 1 study suggested potential benefits with respect to symptoms and cardiac function, the randomized, double-blind, placebo-controlled CUPID 2 trial did not demonstrate any improvement in primary or secondary endpoints [73]. Subsequent studies have shown that this incongruity between the results obtained on animal models and in humans is due to a variety of interrelated factors, including low efficiency of myocardial transduction resulting from poor vector penetration through the coronary endothelium and limited diffusion into fibrous tissue; the high prevalence of pre-existing neutralizing antibodies against AAV1 that could block cell uptake [141]; intervention at a late stage of the disease, when extensive myocardial remodeling and cardiomyocyte loss left insufficient viable tissues for functional recovery [142]. These findings suggest the following provisional conclusion: even a biologically validated target (e.g., *SERCA2a*) cannot succeed without optimized vector design, precise delivery, appropriate timing, and rigorous patient selection.

This translation gap also explains why other promising mitochondrial targets, such as *CPT1B* and *CAV3*, remain the subject of preclinical studies, despite reliable cardioprotection in rodent and large animal models [74,78]. Firstly, neither *CPT1B* (OMIM #601987) nor *CAV3* (OMIM #601253) are established monogenic causes of common HF, as they lack human genetic verification [142]. Secondly, their mechanisms include metabolic restructuring, which carries risks depending on the context: for example, fatty acid oxidation caused by CPT1B can improve energy supply in pressure-overload HF, but at the same time may increase oxygen demand and ROS production during ischemia [143]. Thirdly, achieving therapeutic effects probably requires a high level of homogeneous expression in the myocardium, which requires higher doses of the vector than ion-controlling proteins such as SERCA2a, increasing the risk of hepatotoxicity and immune activation. Finally, after the CUPID 2 setback, this field has strategically shifted towards next-generation AAV capsids (e.g., MyoAAV), immune modulation protocols and targets with human genetics (e.g., S100A1) [144], which has temporarily redirected translational efforts away from complex metabolic interventions. Nevertheless, with advances in precise phenotyping, cardiotropic vectors, and early intervention strategies, these mitochondrial pathways may still find their translation into clinical practice for carefully defined endotypes of HF.

The complexity of HF treatment can also be explained by the peculiarities of mitochondrial functioning—mitochondrial biodynamics, their division and fusion, and mitophagy are complex molecular processes. Therefore, gene therapy targeting one of these components (e.g., overexpression of *Drp1* or *Mfn*) may only exacerbate mitochondrial dysfunction in the context of chronic heart disease.

Based on the identified problems of therapy for HF associated with mitochondrial dysfunction, several future directions can be proposed to address these gaps and limitations. A promising direction is the engineering of AAV capsids to achieve maximum tropism for human CMCs and minimal tropism for the liver; the use of AAV encapsulation in extracellular vesicles may contribute to a reduction in the development of an immune response and an increase in cell specificity during transduction.

Another promising area is the search for and validation of new genes (in addition to the known *SERCA2a*, *S100A1*, *ADRB3*, *CPT1B*) that have defects resulting in mitochondrial dysfunction: oxidative stress, biogenesis, ketone body metabolism, and calcium hemostasis. Genes that promote the transition of CMC metabolism from glycolysis to fatty acid oxidation are of particular interest in this context.

From the point of view of practical medicine, the most important strategy is to determine the stage of HF development at which gene therapy will be most effective: future research should focus on studying the use of gene therapy drugs at different stages of HF, from the initial stages of congestion in the small and large circulatory systems to the final dystrophic phase. Different cardiac diseases leading to HF, such as ischemic, diabetic and hypertensive cardiomyopathies, should also be taken into account.

In the future, gene therapy aimed at correcting mitochondrial dysfunction should be tested and refined in combination with pharmacological drugs already in clinical use (beta-blockers, angiotensin-converting enzyme II inhibitors, diuretics, cardiac glycosides) or in combination with cell therapy. The study of the synergistic effects of all these groups of drugs and approaches is one of the most promising areas of future research for HF therapy.

Although gene therapy for mitochondrial dysfunction in HF opens up new prospects, its path to clinical application requires overcoming significant technical and biological limitations. Strategic research aimed at optimizing delivery, overcoming the immune response, searching for new targets, and conducting thorough preclinical studies on relevant models are important aspects of successfully realizing this potential.

## Figures and Tables

**Table 1:** Molecular targets for AAV-mediated gene therapy of mitochondrial dysfunction.

Model	AAV Vector	Delivered Gene	Mechanism/ Goal	Therapeutic Effect
Mouse model of HF provoked by aortic stenosis	AAV9	*ADRB3* (OMIM #109691)	Prevention of mitochondrial fragmentation, restoration of the mitochondrial dynamics	Hindering the progression of HF
	AAV6 AAV9	*CPT1B* (OMIM #601987)	A decrease in the production of reactive oxygen species in mitochondria	Preventing phenylephrine- induced hypertrophy
Newly born mice with mitochondrial cardiomyopathy	AAV9	*NDUFS6* (OMIM #603848)	A decrease in the activity of mitochondrial complex I, the preservation of the structure of the cristae persisted, and a decrease in the severity of fibrosis	Hindering the development of the contractile dysfunction of the heart
Pig model of HF caused by focal ischemia of the LV	AAV9	*S100A1* (OMIM #176940)	Normalization of calcium metabolism in CMCs, improvement of the endoplasmic reticulum state and energy metabolism	Elimination of both contractile dysfunction and negative force-frequency relationship
Mouse model of diabetic myocardial injury	AAV9	*CAV3* (OMIM #601253)	Restoration of mitochondrial complex I activity and improving mitochondrial function	Inhibition of diabetic cardiomyopathy progression

**Table 2:** Comparative cardiac transduction efficiency of various AAV serotypes across animal models, delivery methods and dosing regimens.

AAV Serotype	Animal Model	Administration Method	Dose, vg/Organism	Key Features
AAV1	Large animals (pigs)	Intramyocardial injection	1.0 × 10^11^	Outperforms AAV2 serotype and exhibits efficient cardiac transduction
AAV2i8	Mice	Systemic intravenous delivery	1.0 × 10^11^	Efficient transduction of cardiac tissue with reduced liver accumulation
AAV6	Mice	Indirect intracoronary injection	0.13 × 10^11^	Highest luciferase activity among AAV1-9 serotypes
	Mice to sheep	Various	5 × 10^11^ vg/kg	High efficacy in transducing CMCs
	Large animals	Intramyocardial injection		Demonstrated high delivery efficiency
scAAV6	Sheep	Percutaneous transendocardial delivery	1.0 × 10^14^	Achieves local transduction rates up to 60% of CMCs
AAV9	Mice	Systemic intravenous delivery	1.0 × 10^11^	High efficacy in transducing CMCs.
	Sheep	Molecular cardiac surgery with recirculating delivery (MCARD)	1.0 × 10^14^	Demonstrates high delivery efficiency
	Large animals	Systemic intravenous delivery	Various (10^11^–10^14^ vg/kg)	Achieves substantial skeletal muscle transduction but limited myocardial expression
	Rodent models	Transvascular delivery	1.0 × 10^11^	Maintains high myocardial transduction activity. The most suitable serotype for rodents
AAV8	Large animals	Systemic intravenous delivery	1–9 × 10^14^ vg/kg	High efficacy in transducing CMCs

## Data Availability

No new data were created or analyzed in this study.

## Abbreviations

The following abbreviations are used in this manuscript:

AAV	adeno-associated virus
APCs	antigen-presenting cells
BCAAs	branched-chain amino acids
CAV3	caveolin 3
CMC	cardiomyocyte
CPT1	carnitine palmitoyltransferase 1
CUPID 1	Calcium Up-Regulation by Percutaneous Administration of Gene Therapy in Cardiac Disease (Phase 1/2)
CUPID 2	Calcium Up-Regulation by Percutaneous Administration of Gene Therapy in Cardiac Disease (Phase 2b)
FAO	fatty acid oxidation
HF	heart failure
HFpEF	heart failure with preserved ejection fraction
HFrEF	heart failure with reduced ejection fraction
IFM	interfibrillar mitochondria
LR	laminin receptor
LVNC	left ventricular non-compaction cardiomyopathy
MCARD	molecular cardiac surgery with recirculating delivery
MHC	major histocompatibility complex
mPTP	mitochondrial permeability transition pore
nAbs	neutralizing antibodies
NOS	nitric oxide synthase
PGC-1α	coactivator peroxisome-proliferator γ coactivator 1 α
PNM	perinuclear mitochondria
PPARα	peroxisome proliferator-activated receptor α (PPARα)
rAAV	recombinant AAV adeno-associated virus
SSM	sarcoplasmic submembrane mitochondria
